# The Impact of Air Inflow and Interfering Factors on the Performance of Microbiological Safety Cabinets

**DOI:** 10.1089/apb.2021.0010

**Published:** 2022-03-15

**Authors:** Simon Parks, Helen Hookway, Kazunobu Kojima, Allan Bennett

**Affiliations:** ^1^ Biosafety, Air and Water Microbiology Group, National Infection Service, Salisbury, United Kingdom.; ^2^ World Health Organization, Geneva, Switzerland.

**Keywords:** biocontainment, biosafety cabinet, aerosol, global biosafety, high-containment

## Abstract

**Introduction:**

The operator protection factor (OPF) of four biological safety cabinets (BSCs) has been measured under standard and suboptimal conditions.

**Methods:**

The OPF for one BSC1, two BSC2, and an acid-fast bacilli staining station (AFBSS) was measured using the potassium iodide method for in situ testing of BSCs (CEN12469) over a range of inflow velocities under standard conditions and with common interfering factors (fans, opening doors, and walk pasts).

**Results:**

The BSC1 and the AFBSS gave a high level of protection under standard test conditions at all airflows (down to 0.3 and 0.38 m/s, respectively). During interfering processes, the BSC1 and AFBSS gave a high level of protection (OPF >10^5^) at the specified inward airflow. At lower airflows, there was a predictable deterioration in performance. There was a significant difference in performance between the two BSC2s tested, with one model passing all tests under all interfering conditions at all airflows. The second BSC2 failed the standard test at the lowest airflow and provided poor levels of protection (OPF <10^5^) in all tests carried out with interfering processes.

**Conclusion:**

Although BSC2s are capable of giving a high level of performance, this is design dependent and the BSC1 and AFBSS give a more predictable level of performance due to their simpler design. In environments where BSC certification is not possible, they may provide more robust and sustainable primary containment.

## Introduction

Open-fronted biological safety cabinets (BSCs) are the most commonly used type of containment equipment in the microbiology laboratory. They are designed to protect the operator, laboratory, and external environment from aerosols generated during the processing of biological samples using directional airflow. They may also have a secondary benefit of providing separation of hazardous activities in a controlled space separate from general laboratory areas. Safety cabinets are subject to a range of international standards for type testing (to ensure that a design performs to standard) and for in in situ/field testing to ensure the performance when it is installed (CEN12469:2000^1^, NSF49:2019^2^). In many countries, cabinets are tested on a regular basis after installation to ensure their performance is still satisfactory, this will be done by suitably trained, and in some cases certified, service engineers. However, in some countries and regions, there is not currently the infrastructure and expertise in country to ensure this is done and hence maintenance can become sporadic at best or may not occur at all. It is also widely known that environmental factors such as cross-drafts, person movements close to cabinets, and opening of doors can affect cabinet performance. Therefore, there may be many BSCs operating in a substandard manner due to lack of maintenance and due to interfering factors.

The Class 2 BSC (BSC2) has become the standard choice of primary containment in many microbiological laboratories. BSC2s are constructed by many manufacturers worldwide to a fairly complex, but fundamentally standard design. However, even within the standard designs, there are many variations. The European standard is performance based and thus allows for a significant level of variation in BSC2 design. The NSF International standard is more prescriptive, specifying a number of discrete design types, reflecting different installation methods and uses. BSC2 can be either recirculated back into the laboratory, connected to the room extract system through a thimble or hard ducted through a dedicated extract system. Although the NSF standard goes into detail on each installation type, the European standard does not, making no distinction in installation types. In essence, a BSC2 provides both inward airflow, to protect the operator, and High Efficiency Particulate Air (HEPA) filtered supply air for the work space, to protect the work from contamination. In broad practical terms, cabinets manufactured to the CEN12469:2000 standard can be considered to be representative of the classified to the NSF49 type A recirculating cabinets. The specified minimum inflow to the A1 is only 0.4 m/s and most European cabinets have a higher inflow >0.5 m/s, corresponding to the A2 type design. The correct operation of these cabinets is a function of both the inflow, driven by the cabinet extract system, and the downflow, driven by the internal blower, if these two factors are not held in balance, then the performance can be significantly compromised. Also, BSC2s can be very sensitive to their positioning within a laboratory and can be affected by being placed close to doors, by other ventilation devices, and people movement.^3,4^ For these reasons, regular testing of the performance of these cabinets is recommended.^5^

BSC1s are designed to be used for applications where product protection is not required. These applications will mainly be cell culture and preparation of polymerase chain reaction reagents. Their design is significantly simpler than the BSC2. Basically, they operate as HEPA filtered fume hoods. This simplicity makes them an attractive option for low-resource countries, as there is less to go wrong, making them simpler to build, maintain, and check correct operation (using vane anemometers). While the U.S. Department of Health and Human Services CDC/NIH *Biosafety in Microbiological and Biomedical Laboratories* 6th Edition (*BMBL*)^6^ describes BSC1, it is mostly in relation to specialist applications. The NSF49 standard makes passing reference to them, but is dedicated to BSC2 cabinets and provides no dedicated test or validation methods. The CEN12469 does describe them in more detail, providing type-specific test methods, which have informed those used here.

In this study, one BSC1, a Class 1-based acid-fast bacilli (AFB) staining station (AFBSS),^7^ and two BSC2 have been tested using the potassium iodide operator protection test from CEN12469:2000 to measure their ability to contain aerosolized particles at a variety of air inflow rates and under interfering environmental factors. The performance of BSC2 cabinets is a balance of inflow and downflow within the workspace, for this study when the inflow was adjusted, the internal downflow across the workspace was maintained, as far as reasonably practical, at the manufacturer's recommended conditions. The potassium iodide (KI) test method was used as it is a mature well-characterized method^1,8,9^ with a long history of use in the United Kingdom, Europe, and internationally. The method was first adopted into the former British Standard BS5726:1992 1992, then subsequently incorporated into the current Europe standard for BSCs EN12469:2000. Within this standard, it holds equivalent status to the biological aperture retention test, which is very closely based on the NSF49 personal protection test and considered to be equivalent to the biological test methods. Unlike biological aerosol methods, it allows tests to be carried out repeatability in a noncontrolled environment and results are obtained directly after each test run, without the need for the 24-h incubation time used for the biological tests. Previous studies have shown that it is a more challenging test than tests using aerosolized microorganisms (Parks, pers. comm.).

In this study, the KI test is used with the standard format but also when cabinets are used under interfering conditions. These are the close presence of a door opening, impact of a cross-draught from a fan, human traffic in front of the cabinet, and the impact of a person working in the cabinet. U.K. guidance^10^ recommends this type of testing when open-fronted cabinets are to be used in BSL-3 laboratories. The BSCs have been tested through a range of inflow velocities to represent BSCs operating suboptimally due to lack of regular maintenance.

## Materials and Methods

### Test Room

The tests were carried out within a nonventilated laboratory used to train staff for deployment in West African Ebola diagnostic laboratories. Tests were performed in the main laboratory space that is ca. 6 m by 6 m in size.

### Cabinets Tested

#### AFBSS

**Figure 1.**
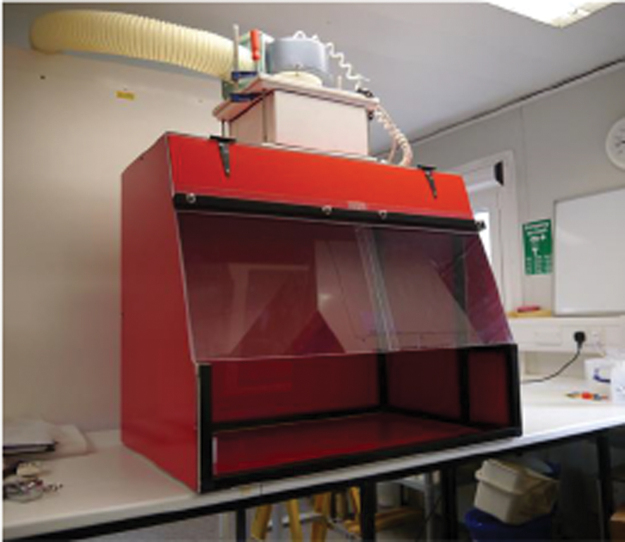
Assembled acid-fast bacillus staining station. Color images are available online.

The acid-fast bacilli staining station (AFBSS) was constructed onsite to a design developed by a partnership with CDC^7^ and described in a document “Ventilated Workstation Manual for AFB Smear Microscopy.” The design uses simple pressed sheet metal to form the body and includes an internal baffle to direct the airflow, as is typical of North American BSC1. BSC1s manufactured to the EN12469:2000 standard do not use this internal baffle, but still comply with the requirements of the standard and provide, when tested using a variation of the methods described in NSF49,^2^ the same performance criteria in terms of operator protection. In these tests, it was decided to simplify the design of the staining cabinet further by the removal of the internal baffle, making the construction easier and making the internal surfaces more accessible for disinfection and cleaning. This cabinet was constructed from a range of simple materials, using hand tools only and was built around a frame made of aluminum box sections. The aim was to prepare a very simple cabinet, which could be easily manufactured, requiring only a terminal fan for operation (Figure 1). The station is designed to be externally ducted, but this was not possible within the test laboratory and its exhaust had to be recirculated within the facility. Hence, to reduce possible cross-contamination between tests, a HEPA filter was installed in-line within ducting, which then exhausted air from the cabinet to a remote part of the facility. The cabinet ventilation was chosen to ensure that the inflow range covering the recommendations from the *BMBL*,^6^ and for NSF BSC1, could be achieved. It was not designed to maintain the inflows described hereunder for a European Class 1 cabinet, but the AFB manual indicates an inflow range of 0.35 to 0.55 m/s, and this was used as the basis for these tests. The inflow was measured in accordance with CEN12469:2000 Annex G.

#### BSC1

A BSC1 manufactured to CEN12469:2000 was chosen as the test cabinet. This U.K. manufactured cabinet has a slopped sliding sash front window and simple internal geometry, in line with the European standard. It is highly representative of the type and design used across the United Kingdom in BSL-3 diagnostic facilities. The cabinet is free standing and recirculates the exhaust air back into the laboratory, through two inline HEPA filters. The BSC1 normal working inflow velocity was 0.85 m/s, when measured using a rotating vane anemometer in accordance with CEN12469:2000 Annex G

#### BSC2(A)

BSC2(A) is manufactured in Europe to the EN12469:2000 standard and the performance of the cabinet has been independently type tested for compliance to that standard. It is a free standing 1.2 m wide recirculating cabinet with a sloped front sash window and was type tested at an inflow of 0.78 m/s. The cabinet has a dual fan configuration, with a dedicated extract fan and plenum to provide inflow and a pair of fans, with their own plenum to provide downflow air. There is a standard ∼30/70 split in the extract/downflow. The average inflow was measured using the manufacturer's instructions, in accordance with CEN12469:2000 Annex G. The inflow is calculated from the cabinet exhaust velocity, as measured at the open extract HEPA filter face.

#### BSC2(B)

BSC2(B) is made by a non-European manufacture to be compliant with the EN12469:2000 standard. The cabinet is 0.9 m wide, with a slightly sloped from sash window, it has a single fan running both extraction and downflow through a single plenum. It has an ∼30/70 split of airflow, with the balance of the flow being controlled by a damper on the extract filter, with total flow then controlled by fan speed. As the extract filter is partly obscured by the control damper, the inflow was measured in accordance with the manufacturer's instructions, using the restricted access method, as described in NSF49 2019 Normative Annex five field tests. The cabinet has an inflow set point of 0.45 m/s.

### Test Methodology

#### Determination of inward airflows for BSCs

The basic airflow requirements for Classes 1 and 2 BSCs from European Committee for Standardization, NSF, and *BMBL* are outlined in Table 1. As the NSF49 standard does not provide performance specifications for BSC1, nor does it provide a test method, the inflow for the Class 1 BSCs and staining cabinet was determined using the methods described in EN12469:2000 Annex G using a rotating vane anemometer.

**Table 1.** Inflows defined in EN12469:2000, NFS49:2016, and *BMBL*

	Class 1 BSC inflow (m/s)	Class 2 BSC inflow (m/s)	Class 2 BSC downflow (m/s)
EN12469:2000	0.7–1.0	>0.4	0.25–0.5
NSF49 Type A2 (Type A1)	n/a	>0.51 (>0.38)^a^	As specified by manufacturer^a^
*BMBL*	>0.38	n/a	n/a
WHO	0.38	0.38–0.51	n/a

^a^ Actual airflows are set by the manufacturer; these values are used for the NSF certification and must then be replicated in the field.

*BMBL*, *Biosafety in Microbiological and Biomedical Laboratories*; BSC, biological safety cabinet; n/a, not applicable.

For the BSC2, inflow was determined using the methods recommended by the manufacturer. In the case of BSC(A), this was undertaken by measuring the volumetric exhaust flow and back calculating the average inflow in line with the EN12469:2000 standard. This was achieved by taking airflow measurements directly from the face of the cabinet extract filter using a rotating van anemometer. The downflow was then measured using the method described in the same standard. For BSC2 (B), airflows were taken using the recommended methods, taken from the NSF49:2019 standard. Inflow was determined using the constricted access method (Normative Annex F), in accordance with the supplied cabinet data plate. Downflows were also measured using the NSF method, using a test grid determined using the standard.

#### Operator protection test

This test was used to determine the operator protection factor (OPF) provided by the cabinet using the potassium iodide method described in BS EN 12469:2000 Annex c.3. The OPF for the cabinet was determined by generating aerosols of potassium iodide within the cabinet and sampling outside the cabinet using the “KI-Discus” Mark II apparatus (Containment Technology Ltd) operated as described in the handbook and standard. For the standard tests, the airflow entering the cabinet was disturbed by introducing a cylindrical arm through the center of the working aperture into the working area to simulate the effects of an operator's arm. A fine mist of potassium iodide droplets, produced by a spinning disk aerosol generator (STAG), fed with a 15 g/L solution of potassium iodide in 80% ethanol, was used as an aerosol challenge. The STAG was either placed above the artificial arm for the BSC2 or below the arm for the BSC1 and AFBSS. In both cases, it was placed 100 mm behind and level with the plane of the front aperture. Four centripetal collectors sampled the air outside the cabinet in defined positions, left and right levels with the bottom edge of the window, and left and right levels with the top of the cylindrical arm. All samplers were placed 150–160 mm from the front aperture and deposited any potassium iodide particles entrained in the sampled air onto filter membranes. At the end of the sampling period, the filter membranes were placed in a solution of palladium chloride (1.0 g/L solution in 0.1 mol/L hydrochloric acid), whereupon the potassium iodide “developed” to form clearly visible and easily identified gray/brown dots. From the number of droplets in the challenge produced by the mist generator and the number of droplets collected in the air samples, the OPF for the cabinet was calculated for each position and an average was calculated.

The value of the OPF was calculated from the expression

OPF = 62 × 10^5^/*n*, where

*n* = the number of spots.

Therefore, the highest performing cabinet from which no particles are released would give an OPF of >6.2 × 10^6^. The performance standard specified in the United Kingdom is that the OPF should not be <1 × 10^5^. This gives a performance in line with the efficiency of a HEPA filter. This is used as the pass criteria for a correctly performing cabinet in the test.

If the counts on the filter are >100, only a section of the membrane is counted, normally a quarter or eighth, and used to determine the total loading. As the counts become progressively higher, the counts become less accurate. If values are >1600, they should be considered as estimates and the limit of detection being 6200. Therefore, OPF values <2 × 10^3^ should be considered as providing an unacceptable level of containment.

#### Test conditions used

Tests were undertaken in triplicate at progressively lower inflow speeds under the following conditions:

- Standard test—as previously described within the EN12469:200 standard.
- Walk by test—as standard test, but with a person walking past the cabinet eight times, at ca. 90 cm in front of the cabinet, as if passing an operator seated at the cabinet front cabinet.
- Door opening test—as standard test, with a full height fridge placed at a distance of 60 cm to the right hand side of the cabinet, such that when opened and closed, it causes some disruption to the airflows around the cabinet. Door opened and closed eight times during test.
- Fan disruption test—a standard desk fan, placed on a low setting and held at height across the room, 5 m away from the front of the cabinet under test. This was operated during test runs, simulating operation of room air conditioning fan.
- Operator working test—the BSC2(A) was also tested with an operator working in the cabinet, undertaking simple serial dilutions, using a variation of the KI test. For these tests, the artificial arm was removed, but the aerosol system left in the same position, while an operator worked at the cabinet. The samplers were then positioned around the operator, as close as reasonably practical.

For all the tests apart for the standard tests, testing was carried out from low-inflow to high-inflow rate. If a test was passed (OPF >10^5^) at a low inflow rate, it was assumed it would be passed at higher inflow values.

## Results

**Figure 2.**
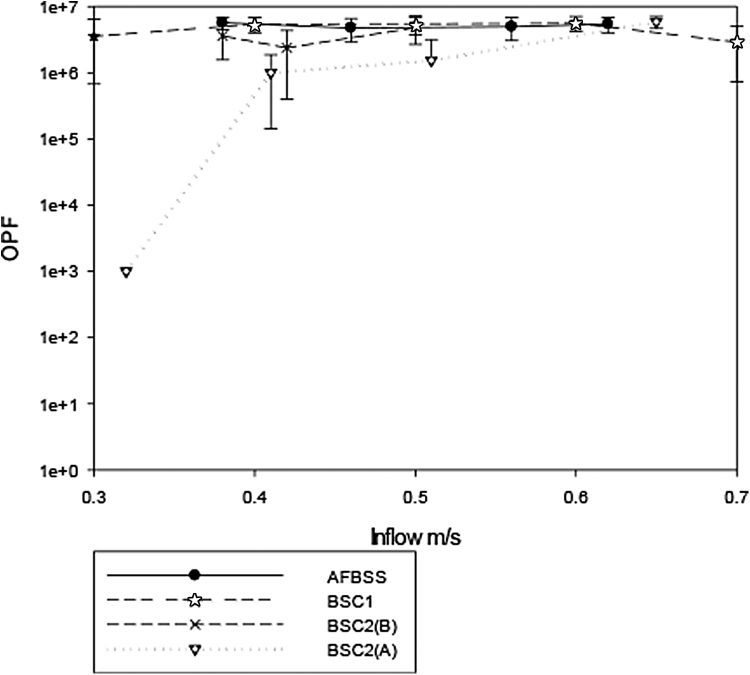
Results of standard OPF testing of cabinets over a range of inflow velocities. OPF, operator protection factor.

**Figure 3.**
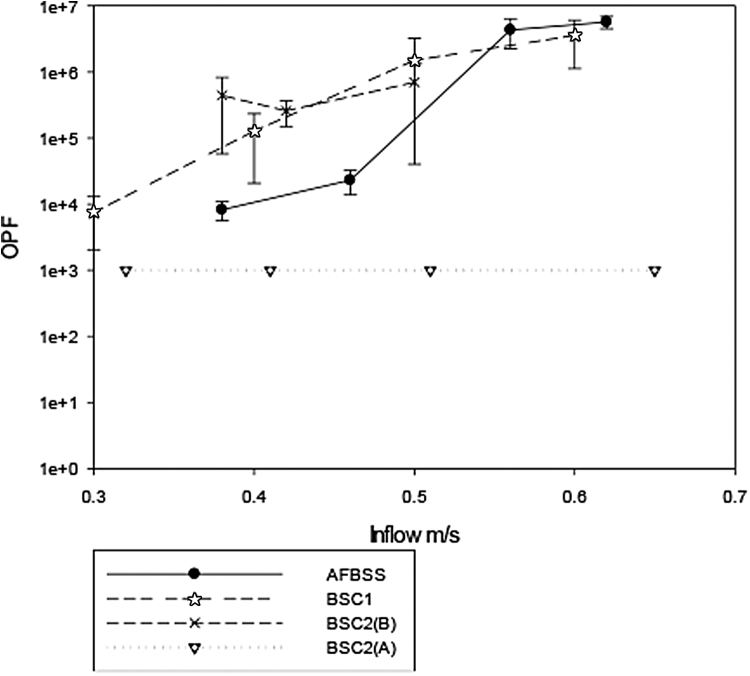
Result of the OPF testing for walk by tests over a range of inflow velocities.

**Figure 4.**
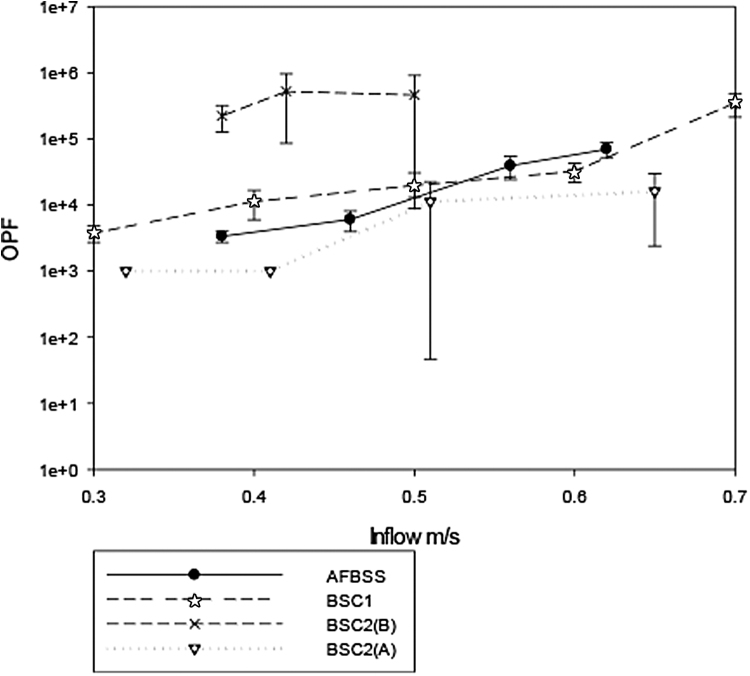
Result of the OPF testing for fridge opening tests over a range of inflow velocities.

**Figure 5.**
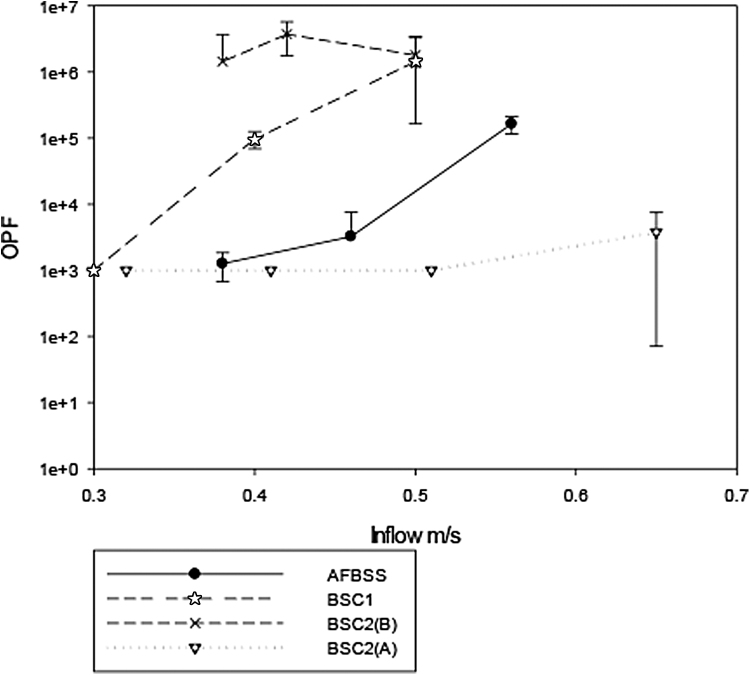
Result of the OPF testing for fan tests over a range of inflow velocities.

**Figure 6.**
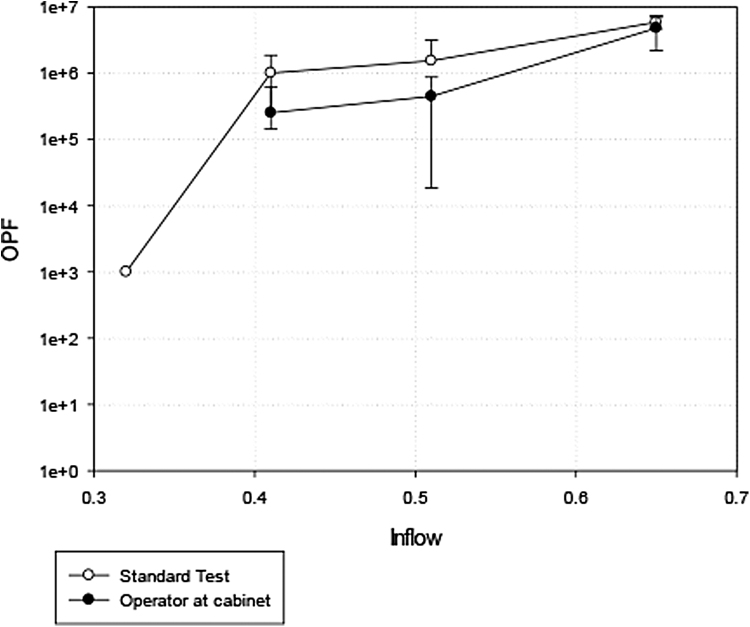
Result of operator working OPF tests over a range of inflow velocities.

The results are shown in Figures 2 to 6.

Figure 2 shows the comparative results on the standard test carried out on all the cabinets over a range of inflow velocities. It can be seen that every cabinet gives a high protection factor for the inflows of between 0.4 and 0.7 m/s. The only test in which an OPF of <10^5^ was obtained was for BSC2(A), wherein the OPF reduces to 10^3^ when the inflow velocity was reduced to 0.32 m/s. In general, BSC2(A) gave the poorest performance through the full range of inflow velocities.

Figure 3 shows the OPF obtained through the inflow velocity range for all the cabinets during the walk by test. BSC2(A) failed all tests at all inflow velocities, including its specified inflow velocity, whereas BSC2(B) passed at all inflow velocities. The AFBSS also passed the walk by test at the highest inflow velocities (0.56 and 0.62 m/s), but the OPF reduced to ∼10^4^ for both tests at the two lowest inflow rates. The BSC1 passed all the tests apart from that carried out with the lowest inflow velocity of 0.3 m/s, where the OPF was 10^4^.

Figure 4 shows the OPF obtained through the inflow velocity range for all the cabinets during the fridge door opening test. Again, the BSC2(B) gave the highest level of operator protection, passing all tests at all inflow velocities. The BSC1 passed the test at its highest inflow rate of 0.7 m/s, and as the inflow velocity was reduced, its OPF gradually declined to 5 × 10^3^ at an inflow velocity of 0.3 m/s. The AFBSS performed in a similar manner with a decline from 7 × 10^4^ to 8 × 10^3^ over its inflow velocity range. BSC2(A) also failed the test over all its inflow velocity range, giving an OPF <10^3^ at its lowest inflow velocities of 0.31 and 0.41 m/s.

Figure 5 shows the results of the tests with an interfering airflow provided by a fan. The BSC2(A) failed all the tests with an OPF <10^3^ at inflow velocities <0.58 m/s. BSC1 passed the tests at the high inflow velocity (0.53 m/s), narrowly failed at an intermediate flow rate, and had an OPF <10^3^ at the lowest inflow rate. The AFBSS narrowly passed at the highest inflow velocity tested (0.56 m/s) and had an OPF <10^3^ at the lowest two inflow velocities.

Figure 6 shows the results when BSC2(A) was tested using the modified standard KI test while an operator was undertaking serial dilutions within the cabinet. All tests were passed down to an inflow of 0.41 m/s velocity with slightly reduced OPF compared with the standard static test with artificial arm.

## Discussion

The standard KI test has been used in the United Kingdom and other countries as a method for in situ testing the performance of safety cabinets for ∼40 years. In this study, every cabinet tested passed the operator protection test (OPF >10^5^) when used with inflows of ≥0.41 m/s. This was the case for the BSC1, AFBSS, and the BSC2s tested in this study. This demonstrates that when cabinets are constructed to U.S. and European standards, they can give a high level of protection to operators under the test conditions even when operated at suboptimal inflow velocity. The results of the in-use tests with an operator carrying out a serial dilution in the poorest performing cabinet shown in Figure 6 demonstrates that BSCs can also perform to a high standard when used to carry out our procedures under normal circumstances under a range of inflow values. However, with interfering factors that may be encountered during use in laboratories, the performance of the cabinets was much more variable especially at lower inflow values.

This is reflected in the U.K. Advisory Committee on Dangerous Pathogens (ACDP)^10^ and *BMBL*^6^ guidance for microbiology laboratories that identifies that BSC2 can be more susceptible to external factors, such as movement within the laboratory and external airflows, than a BSC1. This reflects a belief that due to their more complex internal airflows, the fundamental design of a BSC2 makes them harder to balance and hence more prone to issues around installation. The ACDP guidance makes further recommendation regarding in-use tests for open-fronted cabinets used for hazard group 3 agents. These in-use tests are centered on the KI-Discus operator protection tests, measuring the impact of external airflows and movement of staff and operation of equipment. Even the limited nature of the results reported here reflects that those concerns are clearly well founded. They also reflect the experience of the report's authors, who have many years of undertaking field certification of safety cabinets, including operator protection tests within the United Kingdom.

The results obtained for the BSC1 and the AFBSS are very similar, with the BSC1 performing marginally better. The simplicity of both designs leads to a very similar work aperture geometry, and the even nature of inflow across that opening gives a constant performance. When the inflow from each cabinet was reduced, the OPF obtained during the tests with interfering processes reduced in a direct relationship.

However, the two different designs of BSC2 gave very different results, BSC2(B) was the best performing of all the BSCs tested, passing all tests at all inflows. However, BSC2(A) gave the poorest performance of all the BSCs, failing all the tests with interfering factors even at the highest inflow velocity. This large difference was found even though both cabinets meet the requirements of the relevant national standards and, under all tests, would have met the airflow performance criteria described in CEN12469, for both inflow and downflow. Both have been independently type test certified for performance, and when field tested at the manufacturers recommended airflows, both performed to a high standard. These findings provide evidence for concerns about the performance of BSC2 when subjected to interfering factors, which can significantly degrade performance. But they also show that BSC2 can be designed to give a very high level of containment performance. However, as both cabinets meet the basic design requirements of the relevant standards, it clearly shows that simple compliance to national standards is not a guarantee of robust performance in real-world conditions. There is a small difference in the width of the two BSC2 cabinets, and although anecdotally it has been suggested that smaller cabinet widths may be more robust in performance, this is not reflected in the literature. It is the personal experience of the authors that differences in performance only become apparent with larger widths of ≥1.5 m.

The KI-Discus operator protection test is mainly used in the United Kindom and for specialist high-containment applications in other countries. But it is normal practice to ensure that cabinets meet the performance specification, as determined at type testing or certification to national standards. Often, as with the NSF49 approach, the view is taken that if a cabinet's airflows are balanced to match those of the type tested unit, then equal performance can be expected. However, this is tempered by the need to strictly control the working environment. The safe installation of BSCs is heavily dependent on the location within the laboratory and the impact of laboratory ventilation and work activates. The results from this study further underline that these aspects of cabinet installation and use are critical to safe operation. The NSF49 standard does go some way to address these issues, but the CEN12469 standard fails to do so.

Controlling the operational space within a laboratory is critical to ensure the safe operation of BSCs, this is reliant on both the knowledge of the service engineers installing cabinets and critically the training of the staff using the equipment. All the BSCs tested performed well under optimal conditions even at suboptimal inflow rates.

The simple AFBSS was shown to achieve high levels of operator protection, even at relatively low airflows, compared with those defined in the European safety cabinet standard. Even at the lower airflows specified in the *BMBL* and WHO Laboratory Biosafety Manual^11^ (Table 1), it still achieved very good levels of performance for the standard tests, and outperformed the BSC2(A) under all conditions. Given the simplicity of the design and low cost of construction, it is an excellent solution for primary containment for low-resource countries. Although it lacks basic alarms and control systems, the option to manufacture a BSC from locally sourced components at a fraction of the cost of a certified BSC, which can then be maintained by local engineers, shows clear advantage to purchasing of BSCs from out of country.

The finding of this study strengthens the view that inherently BSC1s tend to be more consistent and predictable in performance than BSC2s, and the ability of operators to carry out regular airflow measurement using basic anemometers is more likely to provide a robust safety performance.

The results of this study show that if BSCs, whether Class 1 or 2, are installed correctly and situated away from doors, fans, and traffic, they should give a high degree of protection to the cabinet operator. The best safety cabinets (BSC2(B)) will give a high level of performance even when not operating at the stated inflow rate and poorly situated in a laboratory (i.e., close to fans, personal movement, and doors). Other safety cabinets will give a good performance at their stated inflow rate even when poorly sited (BSC1, AFBSS). Other cabinets, BSC2(A)s, will give a poor performance even at specified inflow rates when poorly sited.

This suggests that in laboratories where regular cabinet certification is problematic and laboratory conditions may be suboptimal, BSC1s or similarly designed containment devices may be the most effective way to provide primary containment as their inflow velocity can easily be checked on a regular basis. However, for well-designed and controlled laboratories with regular cabinet certification services, BSC2s that have been shown to be resistant to interference would also be a good choice.
